# Bouncing back: a longitudinal examination of challenge within football academy environments

**DOI:** 10.3389/fspor.2024.1402570

**Published:** 2024-07-04

**Authors:** Foivos Papastaikoudis, Rosie Collins, Dave Collins

**Affiliations:** ^1^ Department of Sport, Health Sciences and Social Work, Oxford Brookes University, Oxford, United Kingdom; ^2^ School of Health and Human Performance, Dublin City University, Dublin, Ireland; ^3^ Grey Matters Performance Ltd., London, United Kingdom; ^4^ Moray Hopuse School of Education and Sport, University of Edinburgh, Edinburgh, United Kingdom

**Keywords:** talent pathway, challenges in sport, post-traumatic growth (PTG), longitudinal examination, idiosyncracy, coping skills, preparation and support

## Abstract

**Introduction:**

Although substantial research indicates that challenge plays a big role in the development of talent, little is known of the experiences of young performers as they negotiate and learn from these challenges.

**Methods:**

As such, to better understand the nature and impact of challenge on the Talent Development (TD) pathway, we longitudinally tracked nine young footballers from progressive age-bands (five aged 11 years and four aged 14) as they experienced challenge over a 15-month period using a mixed-methods design. Five semi-structured interviews separated by three months were conducted, and data were analysed via thematic analysis.

**Results:**

Our findings suggested that participants encountered recurrent challenging events, categorised into common/uncommon, planned/unplanned, individual-focused/group-focused challenges. Support for the benefits of challenges was pervasive throughout the data with participants progressing through stages with each challenge (drop, rebound and growth). However, the magnitude and rate at which participants experienced these stages was highly idiosyncratic. Indeed, the perceived impact, scale, and, ultimately, consequential developmental impact of these challenges appeared to vary greatly with participants displaying different responses to similar events. Such differences were underpinned by several individual factors (such as prior experiences and perceived coping skills), contextual characteristics of the challenge (such as type and timing) and support around the challenge (such as priming and reflection opportunities).

**Conclusion:**

Implications include the need for coaches and psychologists to systematically assess and carefully monitor the individual circumstances, needs and skillset of young performers and use this information as a platform for deploying individualised, timely and developmentally appropriate challenges along with relative support to ensure optimal learning and growth from them.

## Introduction

The role of challenge in talent development continues to draw considerable attention. Indeed, there is increasing evidence to support the benefit of experiencing a range of challenges along the TD pathway (1–4). More specifically, recent literature posits that navigating adversity can facilitate growth and development through the process of deploying and refining existing psycho-behavioural skills (5–8). Importantly, however, despite these apparent advances, little is known of the experiences of young athletes *during* the challenge.

An important consideration is the retrospective methodologies used in the majority of studies to date (9). Normally, retrospective interviews are conducted with professional athletes who are asked to reflect on notable past events. Problematically however, longer retention intervals can result in lower accuracy of recall for all types of memory (10). Participants may be inclined to recall only a small amount of vivid and positive memories, some of which may not even be representative of their career trajectory [Brown & Kulik's flashbulb memories, (11)]. Notably, the recall of these vivid experiences is likely to be influenced by the participants' current attitudes and behaviours (12) and, ultimately, their eventual success (13). This stresses the importance of examining the experiences of young performers *as* they negotiate and learn from challenge to enrich our understanding on their role on the TD pathway.

Whilst commonalties experienced by young performers when negotiating setbacks have been observed in previous research, it is also important to consider that the development pathway itself appears to be unique to each individual (14). Indeed, recent findings show that challenges are highly idiosyncratic, with young performers' interpretations and experience of challenge varying greatly (8, 15). As such, the challenge experience is increasingly recognised as highly individual, with athletes showing significant variability in their responses to similar events (4). Resultantly, research has now focused on how young athletes define, interpret and negotiate challenge as they develop (8). For example, young athletes moving into a specialisation phase (i.e., aged 11) are expected to have less experience to draw on to help them navigate some of the inevitable challenges they may encounter than those moving into an investment phase [i.e., aged 14; cf., (16)]. Furthermore, given that the characteristics of the individual may impact the response to challenge (17, 18), it would be important to explore and better understand the idiosyncratic nature of navigating challenges during key transitions on the TD pathway.

In addition, although there appears to be an increasing consensus on the purposeful deployment and preparation of deliberate “bumps” (7) as a means of maximising development, it is also evident that young performers will encounter a variety of naturally occurring challenges as part of their TD journey (19). Whilst some transitions can be anticipated [e.g., normative transition from youth to senior teams; (20)], or strategically implemented by the coaches (21), young athletes may often face non-normative transitions and/or unforeseen events [e.g., sport injury; (22)]. In short, the extent to which growth is experienced as a part of a carefully planned process or a series of emergent occurrences should also be an area of interest.

It is apparent that the adversity-related (often referred to as post traumatic) growth research has much to offer to current TD wisdom. Building from the findings already highlighted and given the established gap in research of tracking performers in real time, longitudinal research seemed to be an imperative lens to explore. Therefore, the aim of the present study was to longitudinally track players as they experienced challenge along the TD pathway using a sliding population approach. Through this, we explored three interrelated features of the players' experiences: (1) the nature and timing of perceived challenges experienced by young footballers, (2) the perceived impact, benefits and subsequent development from these challenges, and (3) the idiosyncratic nature of these challenges.

## Method

### Research philosophy and design

In line with our aim of providing practically meaningful knowledge about young performers' experiences of negotiating and overcoming challenges on the TD pathway, a pragmatic orientation was adopted. This approach encourages selection of methods whose primary focus is to answer questions and offer applicable solutions (23). This philosophical base guided all stages of our research process. As such, we considered that the aims of our study would be best addressed by a mixed methods design (24). Given the nature of research questions which focused on the timing, nature, impact and mechanisms of challenge in young performers’ development, both quantitative and qualitative methods were deemed appropriate. To clarify, mixed-methods research involves the integration of data from both quantitative and qualitative methods to shed light on the same main purpose (i.e., gain a deep insight into the young performers' perceptions of their pathway) (25, 26).

For the identification and selection of information-rich cases related to the phenomenon of interest, a purposeful sampling approach was used (27). This involves identifying and selecting individuals that are especially knowledgeable about, or experienced with, a phenomenon of interest (28). In the context of this study, young footballers currently engaged in the early years of formalised and selective TD pathways were selected. This purposeful sample allowed for deep exploration of experiences of young footballers as they negotiated difficult challenges in the early years of the talent pathway. To this extent, we considered ourselves as co-constructors of knowledge and this study was aided by our applied knowledge, skills, and experience as active practitioners in TD pathways (23, 29). This is consistent with the basic tenets of a pragmatic approach which suggests that researcher biases and preferences can be used to support novel insights (23).

### Participants

Aligning with the aims of this study we recruited participants (*N* = 9) from two Category 1 professional academies in football. Academies in the UK are categorised on a 1 to 4 by the Premier League, with 1 being the most elite (30). As such we recruited participants from those academies viewed as the best resourced and the most effective. Participants were from progressive age-bands (five aged 11 years and four aged 14; *M* = 12.3years, SD = 1.49). Participants were then tracked over a 15-month period. Young performers from progressive age-bands were selected to explore a range of experiences on the football-specific TD pathway. The participants were all male and were selected based upon the perceptions of their experienced talent developer coaches as possessing the most potential to achieve future sporting success. Additional information regarding the different chronological phases of the study can be seen in Table 1.

**Table 1:** Chronological phases.

Age groups	U12s				U13s				U14s				U15s				U16s				U17s
Months	Jul	Oct	Jan	Apr	Jul	Oct	Jan	Apr	Jul	Oct	Jan	Apr	Jul	Oct	Jan	Apr	Jul	Oct	Jan	Apr	Jul
Group 1	0	3	6	9	12	15															
Group 2													0	3	6	9	12	15			

### Procedure

Following ethical approval from the authors' institutional ethics committee (UCLAN Unique Reference Number BAHSS 530), professional sport academies were initially approached and provided with a written proposal about the purpose of the study, the procedures, the timescale (and input required from participants), and planned outcomes. Participants meeting the criteria outlined were invited to participate through personal contact, via their parent or guardian, and informed consent was gained from each participant prior to their first interview. As players were all under the age of 18, a parental assent was required, along with participant consent.

Each participant engaged in five semi-structured interviews separated by three months across a fifteen-month period. Interviews were conducted by the first author and recorded on a dictaphone. Each interview lasted approximately 30–40 min. Guided by the exploratory nature of the study and based on the pragmatic approach (23), a semi-structured interview guide was developed (see Tables 2, 3). The semi-structured interview guide included key open-ended questions that elicited responses informed by appropriate TD literature and our applied experience. Relevant follow-up prompts and probes [c.f., (31)] were also used to encourage expansion on key points (32) and coherence in the depth of responses (33). This format also gave scope to reorder questions and the ability to reroute the interview based on perspectives offered by the participant.

**Table 2:** Interview guide: interview 1.

Purpose	Question	Prompt	Analysis
Examination of pathway to now; key incidents, identified critical incidents.	Using a timeline, can you draw me your pathway to where you are now in the academy?	When did you start and how? What sports did you take up? What did your early experiences look like? What did they mean to you? What were the biggest ups and downs (i.e. sport and/or life challenges)? What were the biggest learning experiences? What did you learn that was subsequently useful? For example, specific skills?? *Using the timeline drawn by participant*: What helped you the most here? *(Pointing to challenging occasions)* Who helped you the most here? *(Pointing to challenging occasions)*	Nature of Involvement Description of early experiences Description of past critical incidents Major critical incidents - stand out as being significant incidents Positive and negative developmental impact of challenges Skills developed through challenges Psychological characteristics possessed, developed, deployed. What skills did they have? Social Support
Retrospective examination of the season so far (i.e., the last 3 months- key incidents, identified critical incidents, reaction to critical incidents).	Can you tell me what happened over the past three months?	What did your experiences look like over the past three months? What did they mean to you? What were the biggest ups and downs (i.e. sport and/or life challenges)? Why were they so impactful (i.e., learning experiences? What did you learn?	Description of critical incidents Major critical incidents - stand out as being significant incidents Positive and negative developmental impact of challenges Skills developed
Examination of specific critical incidences	What were the most difficult experiences or events (if any) you had in the last three months?	Why was it challenging? How challenging it was? What happened? Were you prepared for what happened? What helped you the most here? How? *(Pointing to challenging occasions on the timeline)* Who helped you the most here? How? *(Pointing to challenging occasions on the timeline)* What do you think you learned from it? Can you give me examples? What would you have done differently? Where are you now? Do you think this was the same for everyone on your team?	Nature of major critical incidents Measure and description of critical incidents Positive and negative developmental impact of challenges Did they possess the skills to make the most of the opportunities available? Social Support (e.g., coach, parents, friends etc) Skills developed through challenges Preparation for future events Measure and description of outcomes Individualised vs group-focused
Prospective examination of the next three months.	On this timeline what do you think the next three months are going to be like?	What do you think will happen? Anything big coming up? How well prepared do you feel? Who helped you prepare? How? How do you think you will manage? Can you give me an example? Is this the same for everyone in your team?	Ability to predict and prepare for future developments

**Table 3:** Interview guide: follow-up interviews.

Purpose	Question	Prompt	Analysis
Retrospective examination/review of the last 3 months- forecast v experience (i.e., key incidents, identified critical incidents, reaction to critical incidents).	On this timeline, can draw me your past three months? Let's review what you thought - was it what you expected?	What did your experiences look like? What did they mean to you? What were the biggest ups and downs (i.e. sport and/or life challenges)? Why were they so impactful (i.e., learning experiences? Was it what you expected? Do you think you were prepared for what happened?	Description of experiences. Were challenges correctly anticipated? Major critical incidents - stand out as being significant incidents Positive and negative developmental impact of challenges Anticipation vs Reality Levels of preparedness
Examination of specific critical incidences	What were the most difficult experiences or events (if any) you had?	Why was it challenging? How challenging it was? What happened? What was your initial reaction like? How did this affect you? What helped you the most here? How? (*Pointing to challenging occasions on the timeline*) Who helped you the most here? How? (*Pointing to challenging occasions on the timeline*) What do you think you learned from it? Can you give me examples? What would you have done differently? Where are you now (timeline)? Do you think this was the same for everyone on your team?	Major critical incidents Measure and description of critical incidents Positive and negative developmental impact of challenges Did they possess the skills to make the most of the opportunities available? Social Support (e.g., coach, parents, friends etc) Skills developed through challenges. Measure and description of outcomes Individualised vs group-focused
Prospective examination of the next three months.	On this timeline what do you think the next three months are going to be like?	What do you think will happen? Anything big coming up? How well prepared do you feel? Who helped you prepare? How? How do you think you will manage? Can you give me an example? Is this the same for everyone?	Ability to predict and prepare for future developments.

Data collection was arranged in three parts. At the first stage of the interview, participants were asked to draw their playing career to date on a timeline, highlighting key challenges and perceived critical events along the *X* axis [see (4, 7, 15)]. Participants were then asked to score their relative progression, between 10 and 0 along the *Y* axis, whereby 10 indicated excellent progress ahead of their expectations whilst 0 indicated a comparative failure. Building on this, the second part encompassed a retrospective reflection on challenging events. Specifically, participants were asked to go through their timeline again, this time focusing more deeply on specific perceived critical periods and, importantly, the time between perceived challenging experiences. This included psychological challenges experienced, initial responses, coping methods employed, significant other/coach inputs and lessons learnt. Finally, the third part explored their ability to predict and prepare for future developments, with participants being asked to consider what was likely to happen over the next 3 months and their level of preparedness for this.

Follow-up interviews employed the same structure as the first but with an additional focus on the previously self-identified challenges followed by their preparedness for future challenges. Three-months was selected as the time frame between interviews as this offered enough time for participants to experience different challenges organically, whilst still being frequent enough to enable accurate recall (34).

### Design and analysis

#### Quantitative analysis

First, a quantitative analysis was undertaken to calculate the impact of these challenges on perceived *progression level*. In doing this, each grid on the graph was taken as 1 unit. The impact of each challenge was calculated by (a) taking the vertical distance between the point where the challenge encountered and where the following decrease or increase in perceived progression reached its lowest or highest point, and (b) working the percentage change in perceived progression level against the level at the time the challenge occurred. To clarify, if a challenge was followed by a drop of 2 units from a perceived potential of 6 units, then this had a magnitude of 30% (i.e., 2/6 × 100 = 30%). Also, if the perceived progression pre-challenge was at 5 units and the subsequent point post-challenge was 7 units, then this was calculated as having a magnitude of 40% (i.e., 7/5 × 100 = 140%; thus 40% increase on their pre-challenge perceived progression level). Similar process was followed to calculate “rebounds” and, more specifically, the percentage increase in perceived progression level from the lowest point following a challenge to the highest immediate point that followed. Notably, the process of calculating relative percentages allowed for comparisons across participants.

#### Qualitative analysis

Qualitative analysis was conducted to identify factors perceived to have helped participants handle and recover from challenge. More specifically, it allowed us to explore the idiosyncratic nature of the challenge and its impact on each particular player. All interviews were transcribed verbatim, then checked for accuracy against audio recordings. Data were then analysed using thematic analysis (TA), selected as it allows researchers to examine patterns of shared meaning across data sets (35). Additionally, a core feature of TA is the recognition that the researcher plays a key role in the process of generating themes through engagement with the data (36).

Interview transcripts were analysed predominately via inductive TA; a bottom-up approach focused on deriving codes and themes from what is in the data (37). In doing so, Braun and Clarke's (38) six-phase approach was employed. More specifically, the first phase involved familiarisation with the data through highlighting areas of interest. Second, codes were initially generated on a semantic level followed by a multiple sweep analysis which allowed for underpinning assumptions to be drawn (35). After identifying and organising themes from the initial coding process, the fourth phase included the process of reviewing and refining themes when appropriate. The fifth phase involved defining and naming themes whilst the final phase focused on producing the final report of data. Importantly, however, this process took place flexibly, with appropriate non-linear movement between phases (39).

### Addressing trustworthiness

Alluding to previous compelling evidence suggesting that trust and rapport can shape the process and outcomes of interviews (40), all interviews were conducted by the first author. Notably, the interview process was further facilitated by the author's knowledge and appreciation of the challenging nature of the TD pathway due to their current involvement in TD settings. Several measures were also taken to ensure trustworthiness. The use of qualitative software (QRS nVivo 9) enhanced the trustworthiness of the data analysis process. Additionally, on completion of data analysis after each interview, interview transcripts were returned to each participant to encourage member reflections (41). In doing so, participants were sent their analysed transcript via email, asked if the identified themes reflected their own experiences of navigating challenges and if they wished to share any additional information. This process was further facilitated by a 10–20-min face-to-face conversation with each participant in the first part of the follow-up interview, allowing important time for them to reflect on their experiences in greater depth. In fact, all participants engaged in member reflections at some point during the 15-month period. Their additional reflections were then incorporated into the results, thus, enhancing the richness of the overall analysis.

Additionally, in accordance with the pragmatic approach where the researcher is considered an active agent, the lead author maintained a reflexive journal. This approach was adopted to reduce the likelihood of interpretative bias (33) as well as critically consider the methodological approach (42). Finally, to ensure resonance in our approach, the first author's emerging interpretations were challenged by the second and third authors who acted as critical friends (43). For clarity, the second and third authors reviewed the annotated and coded transcript. Where alternative coding was suggested, reflective discussions (44) took place to address alternative explanations, until mutual agreement between authors was reached.

## Results

The aims of this investigation were to explore (1) the nature and timing of challenges perceived experienced by young football players, (2) the perceived impact—benefits and subsequent development and (3) the idiosyncratic nature of these challenges. The results begin with an overview of the participants' perceived challenges, as displayed in Tables 4–6. Secondly, we present exemplar progression graphs drawn by participants across a fifteen- month period. Thirdly, we present excerpts from the participants' timelines to illustrate the idiosyncratic nature of navigating specific challenges. For clarity, these results were developed from the data collected in all five interviews. All themes are presented in the results section with exemplar quotations to illustrate the analysis, as well as the percentages of participant reporting each theme. These percentages are displayed to demonstrate the frequency with which participants offered certain responses. They are not intended to display any differing importance or value of the findings.

**Table 4:** Common and uncommon challenges.

Higher order theme	Lower order theme	Exemplar quotes
Common 100%	Playing up 100%	“It was hard, it was more physical. I know I’m not stronger and faster than them, so I need to just be smarter than them. You need to make quick decisions; you have to be focused.” P9
	Failing to achieve team targets 100%	“This year we thought that we could win this because we did so well last year. I'm not so sure what it was, we just couldn't score. We’re all very annoyed with ourselves…We could’ve done more but we didn't.” P2
	High-level competition 100%	“It was a tough game. Because there's lads that are a lot stronger, taller, maybe quicker. You need to adapt your game, maybe give them two yards. One of their midfielders is good. It was tough trying to mark him.” P6
	Transition to a higher age group 100%	“It is definitely the pressure to perform well, like to be consistent and show that you are taking it seriously.” P7
	Underperformance 100%	“The most upsetting moment was in Holland. It was the quarter final of a tournament. We went into penalties, and I missed a penalty. When you feel we could have won it, we were so close. That was hard.” P6
	Injury 78%	“When you can't play football it's frustrating and it feels down. Frustrating as I have only been here for Physio. You are missing out on a lot of things, like big tournaments and games” P1
	High workload 78%	“I think sometimes, mixing schoolwork and football is draining. So, if you have a hard work, training and matches, then you have schoolwork on top of it. It's tiring and draining and you’re not in good mood.” P3
	School exams 67%	“We have had exams last week in school, just to see where we’re at with our learning. It felt scary, but then once you got in it, it was much easier than everyone predicted it to be.” P9
	Changing school 56%	“Leaving Primary school was quite hard. You know leaving all my friends. A bit scary for me” P8
Uncommon 100%	First national team call-up 44%	“You’ve been selected as one of the twenty best in your age group to play for your country, you’ve got to prove it as well. It can be a statement, but you have to do your best. Pressure is on.” P4
	Playing out of position 44%	“I think it was tough in the beginning because my mind is very defensive; it still is now, I always think ‘shall I go back?’. But I just need to learn to get the attack in my mind instead.” P6
	Living at digs 44%	“As soon as I joined digs, I felt like I missed home a lot. Throughout the year, I missed home a lot.” P3
	Growth spurt 44%	“I have growth pains, like my bones hurt. Some days I feel better but I’m not playing well. I’ve missed a few training sessions; a bit up and down you know.” P9
	Pre-season training camp 44%	“I went to Poland for a pre-season in July. It was quite tiring because we had to wake up early in the morning and go for a run. Then multiple sessions and very high intensity. It was a new challenge.” P3
	Bereavement 33%	“There's just a lot to get through, isn't there? I’d say it's mostly just family passing away, because there's been a lot of that recently. You know they won't be around anymore and it's sad.” P4
	Missed selection 22%	“You’re working hard… you are giving your best day-in day-out. You’re waiting to be called up and then you realise it isn't going to happen. You don't like it, it's pretty frustrating.” P4
	Personal illness 11%	“When you are so young, it is quite scary, you feel worried. Is it going to be hurtful, is it serious? How long I won't be playing football for? Will it affect my development?” P5

**Table 5:** Planned and unplanned challenges.

Higher order theme	Lower order theme	Exemplar quotes
Planned 100%	Playing up 100%	“It was well-planned and thought by my coaches. They explained what the expectations were and how they would support me throughout.” P8
	Pre-season camp 100%	“Definitely a big part of getting you ready for the season. Very demanding, training is intense. I think the coaches know how to get you ready for the season” P2
	High-level competition 100%	“We are winners, we always have the ambition to win big tournaments. And obviously, the coaches want us to have this mindset. We need to get used to this pressure if we want to become pro” P9
	High workload 100%	“Everyone's been very supportive. The coaches, teachers, welfare staff. It is obviously hard to balance it. They are doing whatever they can to support us” P3
	Transition to a higher age group 100%	“It is a different level. Higher expectations, higher level of competition, more pressure. Support from people at the club has been great, trying to give us the best opportunity to succeed.” P1
	Living at digs 100%	“I stay in a house with another boy. I thought it's going to feel harder to move, but the club made it so easy, you feel safe around this environment. The coaches and well-being staff are nice, and our housekeeper is attentive.” P1
	School exams 100%	“The teachers know how stressful it is, but we also know education is important. They are doing the best to help us. Like feedback, what we need to focus on and things like that” P7
	Missed selection 100%	“I mean…the coaches want to push me more… They know how determined I am, and how much I want it. They challenge me, they want to see even more from me before they play me up.” P4
	Changing school 100%	“My Mum's words have always been reassuring. But I did consider asking about homework. My brother had just left this school, so he helped a lot. He gave me equipment, like a scientific calculator; things you do need in secondary school.” P9
	Playing out of position 75%	“I think the coaches want to stretch me a bit. The want me to develop some other skills that will make me a better footballer.” P6
	Failing to achieve team targets 100%	“You never plan for it. It was one of our team targets… I don't know. There needs to be a Plan B, how to react and how to get back on track quicker.” P2
Unplanned 100%	Injury 100%	“It does happen a lot, but you don't want to think about it … Obviously, you don't want it. And of course, you can't plan for it.” P6
	Personal illness 100%	“We only found out a month ago. It was sad. When you’re so young, you don't expect it, you’re not supposed to have these problems.” P5
	Bereavement 100%	“These things happen but you are never ready for them. It's a shock, it's a massive loss” P4
	Underperformance 78%	“You never want to perform badly; you don't want to let yourself down.” P7
	First national call-up 75%	“Well, I wasn't thinking about it, so I’m super excited! Everyone wants to play for their country. It's a good surprise… Especially when you’ve not experienced it before, it's a new challenge.” P1
	Growth spurt 75%	“Everyone knows is normal, but it is somewhat hard to accept…Especially when it comes at the wrong time, when you have the chance to show everyone who you are” P9

**Table 6:** Individual-focused and group-focused challenges.

Higher order theme	Lower order theme	Exemplar quotes
Individual-focused	Playing up 100%	“Well, obviously other players occasionally play up. But at different times, maybe sometimes in pairs. But this is focused and tailored against my individual needs and development.” P5
	First national call-up 100%	“The scary thing is that you are somewhat alone to this. You are going to a place where you don't know people and you have to prove you are good enough to be there.” P1
	Injury 100%	“Most likely, everyone will experience some type of injury at some point. But everyone's different, their circumstances are different… And the whole experience will be different too.” P9
	Underperformance 100%	“I felt like I let the team down. Everyone was saying ‘it's alright, it's going to be fine’. But it is tough, you feel responsible for what happened. Like the spotlight is on you” P7
	Personal illness 100%	“So, there is more serious things, like more serious illnesses. This age, it is rare which makes it a very personal challenge. It makes you stronger” P5
	Bereavement 100%	“It just feels like nobody can help you really. You have to deal with it yourself.” P7
	Missed selection 100%	“Everyone's got different ambitions. This is pretty unique to me, I’m pushing hard; it's all going to go to plan. I try to focus on me and what I need to do to make it happen.” P4
	Playing out of position 100%	“They do it quite often, the coaches say it is important to learn different moves and understand the game better. But it's slightly different, everyone's got different needs.” P3
	Growth spurt 100%	“I know the other lads are going though it too as we’ve been discussing about it. But for everyone's different… different times, different pain.” P8
	School exams 100%	“I don't know whether the other boys see it as a challenge at all. Personally, I do care a lot about my education.” P9
Group-focused	Failing to achieve team targets 100%	“We’re all gutted. We wanted it so much. On our way back to hotel it was complete silence.” P8
	Living at digs 100%	“Well, I can't speak of others, everyone's experiencing it in a different way. But I know that all boys sort of struggled in some way. We look to try and support each other.” P1
	Pre-season training camp 100%	“It's hard for all of us. We discuss this quite a lot, it's definitely a different level. Sessions are a lot more intense… expectations are higher. Things are getting more serious.” P3
	High workload 100%	“Yeah, I guess managing this can be tiring for everyone in the team, especially those on the full-time programme. It can be overwhelming at times.” P4
	High-level competition 100%	“It should be the same for everyone. We need to be physically and mentally ready for these games. We have a common goal. Don't know how seriously everyone's taking it though.” P3
	Transition to a higher age group 100%	“It is a step up for everyone, or at least it requires everyone to keep working hard. It becomes more challenging for all of us.” P6

### Nature of perceived challenges

The participants identified numerous aspects of their progression on the talent pathway as challenging, with these challenges falling into three categories; common vs. uncommon, planned vs. unplanned and individual-focused vs. group-focused.

#### Category 1: common/ uncommon

As per Table 4 a difficult challenge consistent amongst all participants was being selected to *play up* an age grade. Whilst they described it as an exciting developmental opportunity, P8's account spotlighted the emotional impact this challenge can have, “I was happy as I felt the coaches praised me for my performances. But I was worried at the beginning. A bit out of context. The level was different, players were moving faster and playing the ball quicker. Tough but a great opportunity to grow”.

Participation in *high-level competitions,* such as domestic and/or European tournaments, presented a significant challenge for all. In fact, they recalled the intense levels of pressure to perform at these events whilst facing the possibility of success and failure. As P1 described “You feel the pressure to show who you are and what you can do. That's what you have been training for. Hours, days, months of preparation for these moments.”

All participants identified one of their most difficult challenges as the *transition to a higher age group.* Participants reported facing increased competitiveness, different level of play and higher expectations. Interestingly, participants interpreted the impact of this transition as performance anxiety, worry and uncertainty. Likewise, *failing to achieve team targets* was discussed by many. In fact, participants' accounts clearly outlined the levels of disappointment, frustration and rumination experienced when their team fell short of these expectations.

*Underperformance* was another challenge that all players referred to within their interviews. Notably, this was discussed in different ways, with some players reported struggling with a period of performance slump whilst others reported experiencing it as a single occurrence. Poor performances were often linked to other challenges, such as playing up, transition to a higher age group and high-level competitions.

Not surprisingly, the majority of participants reportedly encountered an *injury* at some point during the course of study. As shown in Table 4, the perceived impact of injury was of a loss of training and competition exposure, with participants displaying various emotional responses such as uncertainty and worry about their progress. Additionally, the participants identified the increasingly *high workload* across multiple domains including football and school as particularly challenging. The perceived impact was an overload of demands, with participants describing it as mentally and emotionally taxing. Less frequent among common events were the challenges of *school exams* and *changing school*.

Within uncommon challenges, participants described the difficulties experienced when they had to *play out of position* and the emotional upheavals resulting from this. Moreover, participants identified their *first national team call-up* as significantly demanding, highlighting the perceived pressure to prove themselves and the fear of the consequences of not meeting these expectations. Albeit less reported overall, this challenge still appeared to be particularly common amongst the older participants. Similarly, *living at digs and pre-season training camp* reportedly posed difficult challenges to all older participants whilst *growth spurt* was mainly reported by younger participants.

Interestingly, *missed selection* was only mentioned by two participants. This may be because the participants we recruited were all “highflyers”. As stated earlier, selected by TD professionals as those most likely to be successful in the future. Notably, however, participant accounts implied high levels of emotional impact, with repeated references being made to the levels of frustration and worry experienced as a result of this challenge. Indicative of the individualised nature of the pathway, *bereavement and personal illness* were less evident throughout the data.

#### Category 2: planned/ unplanned

The data revealed a combination of a series of planned and emergent occurrences on the participants' pathway. Planned challenges involved normative events and/or carefully planned obstacles, strategically designed by the coaches to stretch the participants' physical, cognitive/mental and/or technical abilities. In contrast, unplanned challenges presented unforeseen and/or naturally occurring events that participants were unable to prepare for.

As shown on Table 5, experiences such as playing up, playing out of position and transition to a higher age-group were commonly identified as planned challenges whilst injuries, personal illness and bereavement were described as highly unpredictable events. Within planned challenges, P5 reflected on the proactive steps taken by the coach to ensure clarity and optimal preparation for a difficult challenge:

The coaches spoke to me and my dad before I played up with the 13s. They said it's going to be tough but good for my development. Like stretch me mentally and physically. But they've been supportive, like checking in on me and giving me feedback regularly.

On the other hand, P2 described the elements of uncertainty and worry that underpins an unforeseen challenge such as failing to achieve team targets:

It was a shock, no one's expecting this. I thought we were not mentally prepared for this. We are a top team and everyone wants to beat us. We lacked intensity; we were so lethargic. Maybe some thought it would have been easy? It's disappointing.

This distinction becomes less evident across some challenges, further illustrating the complexity of the nature of challenges reported. To clarify, whilst some normative challenges were discussed, particular aspects of the experience such as the timing of occurrence rendered them unpredictable. The following excerpts exemplify this by highlighting the contradicting views on whether growth spurt constituted a planned or an unplanned event. P6, for instance, highlighted how educational workshops provided by their club equipped them with knowledge on how to cope with growth pains and physiological changes when they occur. “Obviously it's part of our nature. Educational bits were helpful, they put a structure to it.” Despite this element of preparedness, however, most participants identified growth spurt as an unforeseen event due to them being unable to predict the exact timing of when this would occur. P8 stated, “It's always different when it comes. You don't know when it's going to happen and how it will affect you”. In short, the unforeseen nature of the timing of some of the above events further alludes on the complex reality of TD environments.

Additionally, although underperformance was commonly identified as an unplanned event, 2 participants seemed to hold a different view. Among others, they raised the fact that poor performance can often be linked to artificial challenges such playing up or out of position. The following quote, from P4, epitomizes the complexity of this distinction:

Obviously it is not always straight and easy, there's lots of ups and downs. The coaches want us to fail so they can see who's got the mindset to get back up. Challenging practices, high intensity and pressure to perform. I mean, you won't play well all the time. So I guess, it is very much structured in that sense.

#### Category 3: individual-focused/ group-focused

Reflecting the nature of football as a team sport, results revealed that participants encountered individual challenges as well as challenges as part of a group. Notably, despite a number of the team challenges were extensively discussed by all participants, table three shows that the majority of them were identified as individualised experiences. To support this view, the participants alluded to different aspects of the challenges such as timing, individual needs, and subsequent impact. This is clearly illustrated by the following quote, from P4:

I think it's quite unique to me… We all have our highs and lows. Everybody is different though. More or less, everybody's on a different journey. Everybody's goals and needs are going to be different so that effects it as well.

Interestingly, even when challenges were designed to impact on a group-level, participants maintained that there were elements which remained highly individualised. For example, P1's quote portrays a sense of ambiguity on the impact of a group-focused challenge across participants, “Everyone's different, maybe others really enjoyed the fact that were moving to the digs. I personally found it hard, I felt very homesick initially”. In short, although some challenges seemed to be occurring at the same time for different participants, it was asserted that everyone experienced them differently. Clearly, the above data are supported by the participants' timelines presented in the following section.

### Perceived impact- benefits and subsequent development

**Figure 1:**
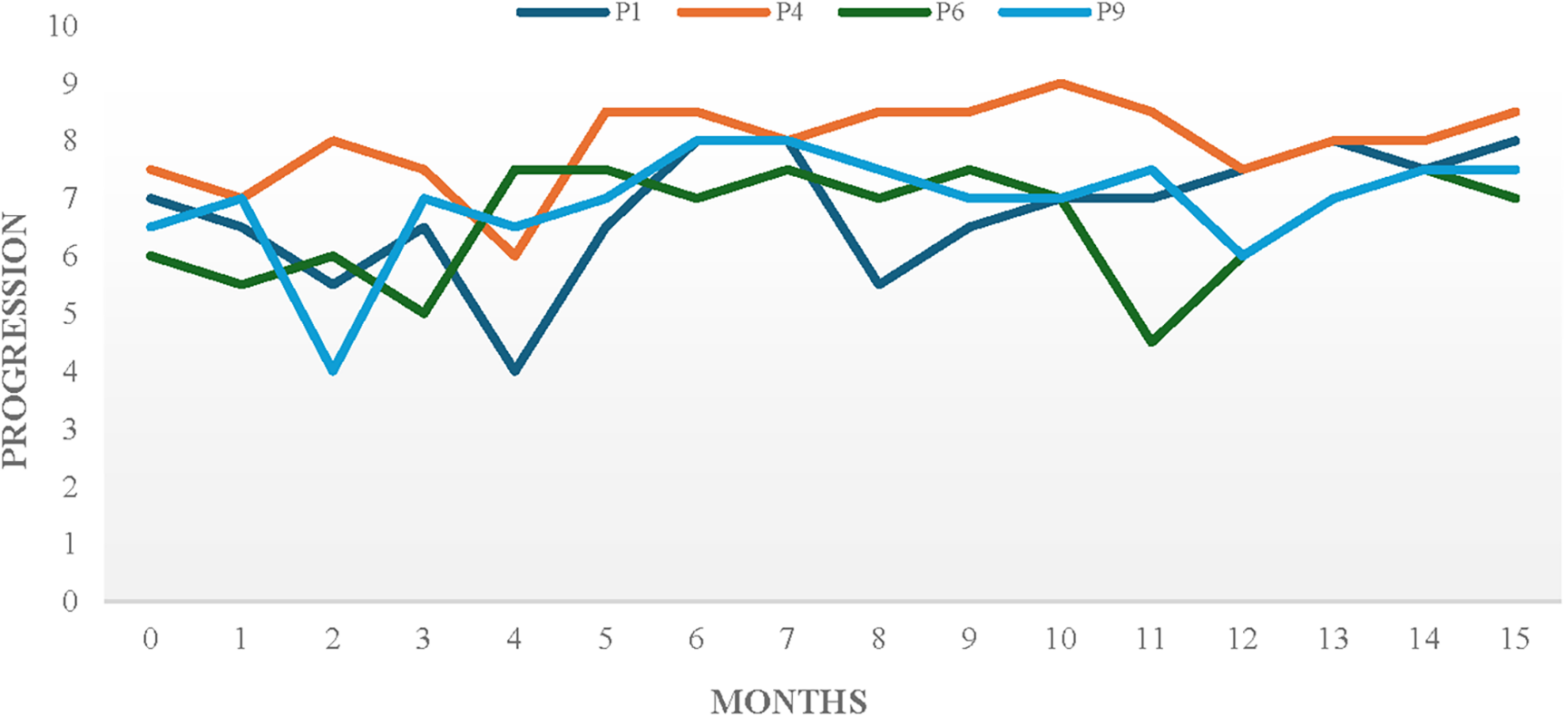
Four exemplar timelines showing progression across a 15-month period.

For all young participants in this study, the pathway was filled with recurrent challenging events (planned/unplanned, individual-focused/group-focused). Notably, all their timelines indicated that the perceived progression level at the end of the study was always greater than it was when it started. Examples of 15-month timelines are shown in Figure 1.

Support for the benefits of experiencing a range of challenges was pervasive throughout the data. Results indicated that challenges were usually followed by an instant drop in perceived progression level. Subsequent rebounds were similar or higher. Typically, however, it was observed that the larger the negative impact the bigger the resulted growth. Notably, events that were followed by little or no perceived drop represented challenges to be “coped” with, thus offering little new learning.

**Figure 2:**
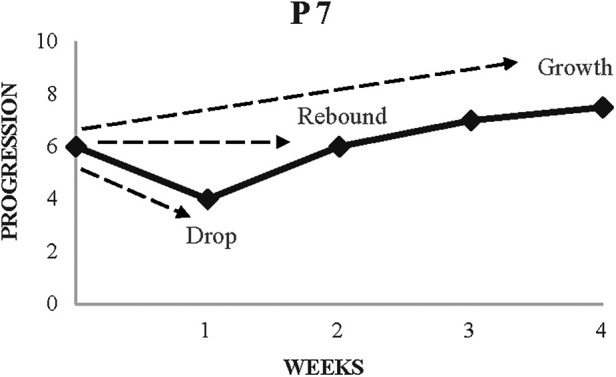
Exemplar timeline showing stages of navigation of a common, planned challenge (playing up).

As shown in Figure 2, participants progressed through a series of stages in each challenge including *drop*, *rebound* and *growth*. *Τ*he drop stage typically involved participants' initial response which was characterised by a variety of emotions such as worry, frustration and uncertainty. For example, P7 outlined “However hard I worked, however much effort I put into it, it wouldn't come off. It's frustrating because you really want it to work, but it doesn't. I hope my coaches don't change their mind about me.”

The rebound stage involved participants bouncing back to the perceived progression levels prior the challenge. In doing so, they appeared to reflect on their current beliefs and explore the challenges with a problem-solving approach before taking a form of action to recover from the challenge [cf. (18)]. For example, P4 described the steps taken to recover from a period of underperformance when he was playing for his national team, “I realised that I was too comfortable, maybe overconfident. This attitude would get me nowhere near where I want to be. It wasn't good enough.” After finding meaning and undertaking belief change through reflection, he added, “I decided to step it up a lot, by watching different videos, writing things down, watching my teammates. It's started to work; I understood my teammates more. Knowing their movements, you can think about what you’re going to do next*.*”

Whilst this stage was clearly associated with initial steps of change, the growth stage involved the participants having reflected and identified benefits and positive changes from the challenge. As per the example below where P3 described the lessons learnt from his experience:

I learnt to focus on myself. So, say, I found it hard going into an older age group and have them shouting at me for losing the ball. I learned to leave that aside and not focus on them, don’t let them distract you. I think that’s helped me through my career so far, to be able to block out people and distractions. I think that's been a big part.

### Idiosyncratic nature of challenges

Despite the common patters of growth discussed above, the magnitude and rate at which participants experienced these stages was individualised. More specifically, the perceived impact, scale and, ultimately, consequential developmental impact of these challenges appeared to vary greatly. Indeed, participants displayed variability in their responses to similar events. These discrepancies were often underpinned by differential interpretations of the impact of the challenge. Indicative of this is the common challenge of playing up. Data revealed that the perceived impact of playing up involved physical mismatches, higher skill levels, social challenges or a combination of them. The following excerpts illustrate how participants were affected differently by this challenge. P7 stated:

I played up for 2 weeks. I was happy because everyone wants to play up. But I struggled at the beginning, especially with the physical side of the game. Players were bigger, stronger, and faster. They were getting their body and limbs across you.

Whilst P7 asserted that playing up mainly posed a physical challenge which then created performance anxiety, P3 highlighted the social challenges involved in his experience:

There is an individual in the age group that everyone in my age group dislikes because he isn't nice. I think there're a few players who make it harder for the younger ones. It's hard to get through sessions because they kind of pick on the younger ones in training.

**Figure 3:**
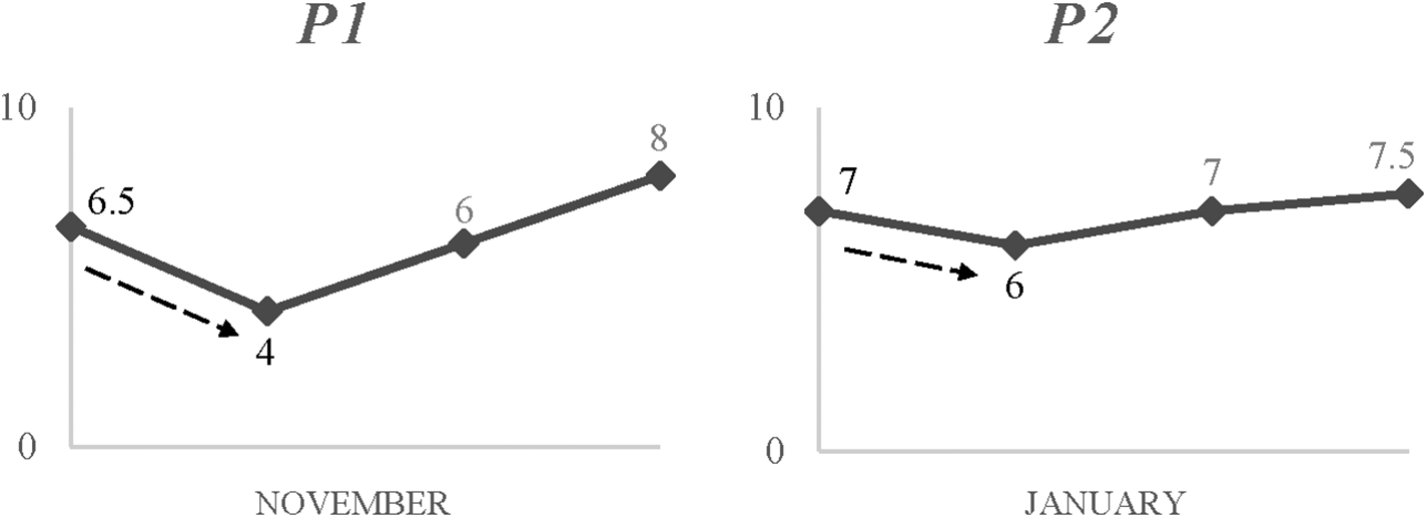
Two contrasting timelines showing impact of a common, unplanned challenge but at different times (injury).

As mentioned previously, participants often faced similar challenges but at different times. Importantly, the variety of responses (i.e., perceived drop) with regards to the scale (i.e., difficulty) and subsequent emotional impact of the challenge appeared to be affected by the timing of the occurrence. As depicted on Figure 3, the perceived drop reported by P1 (38%) following a mild ankle sprain injury was clearly greater than P2's (14%). Notably, however, P1 had only returned from an injury which required two months to recover, “I was injured and that was a downer. I missed the US trip which was a big event. I was really frustrated and sad about that.”. Ahead of his return to training, P1 described his eagerness to play in the upcoming Premier League tournament, “I am now back in training and can't wait for what's coming up.”. Following his re-injury, he recalled the levels of frustration and upset experienced, “It was difficult because I missed another big tournament, so it was mentally too hard. It's so frustrating”. In contrast, P2's trajectory prior to this injury was not as “bumpy”. Despite the inherent frustration felt, he expressed a relief for not missing important events, “It wasn't too serious, so I was lucky. Obviously, it's still frustrating because you don't play but you’re not missing important things so that's good. It's one of those things you have to deal with”.

**Figure 4:**
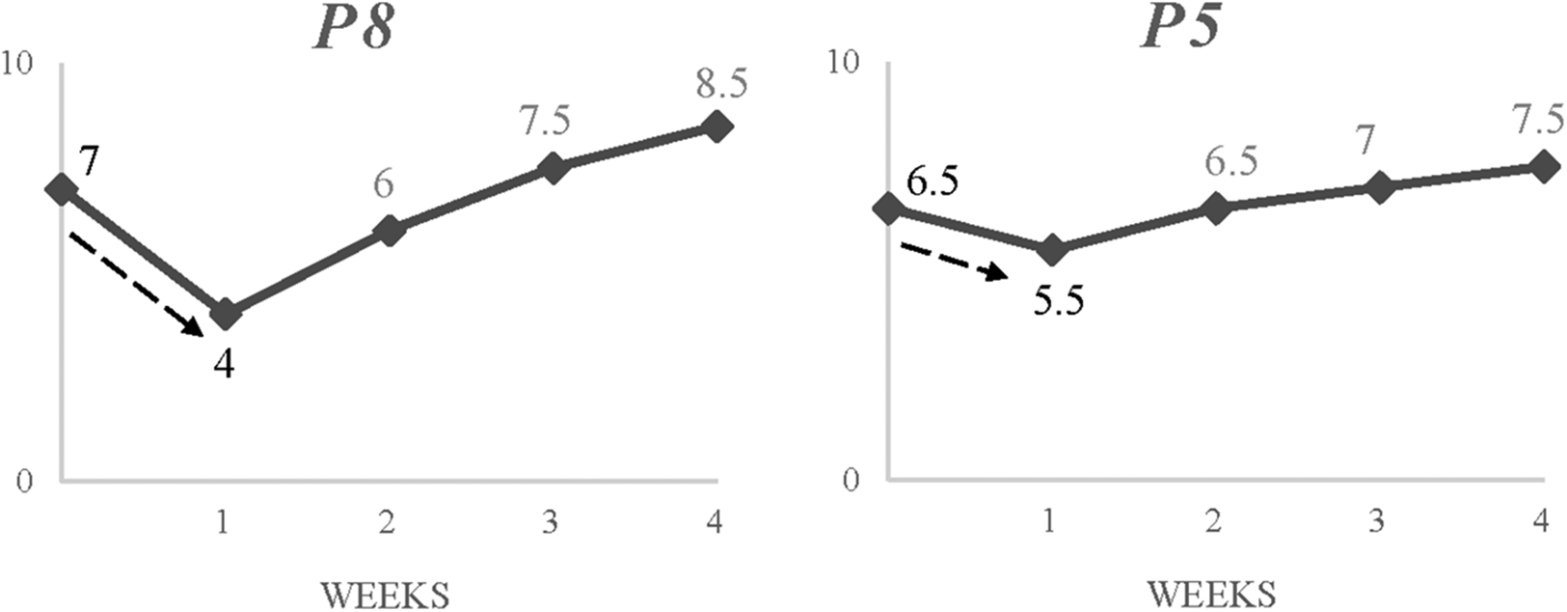
Two contrasting timelines showing impact of a common, unplanned challenge (failing to achieve team targets).

Discrepancies in interpretations and emotional responses were also evident in how participants encountered concurrent group-focused challenges. Figure 4 portrays the different impact with regards to perceived drop the challenge of not accomplishing the team's targets had on different members. P8 (42%) described the level of upset and rumination experienced following the event, “We lost in the final in Portugal. I felt very down. It was a big tournament. We’ve never been so close…I can't stop thinking about it. The hardest moment in my football life*”.* On the other hand, P5's reaction and reported drop (15%) was less severe, rationalising and framing the challenge against a prior experience, then taking a future-oriented approach:

It's tough, but this is football. It's not the first time we have experienced this. Obviously, after my surgery (illness) I realised that these things are small. There's going to be more opportunities to win tournaments. It's more about what you learn.

This clearly suggests that the characteristics of the individual, including prior experience and perceived coping skills, can impact the initial response to challenge.

**Figure 5:**
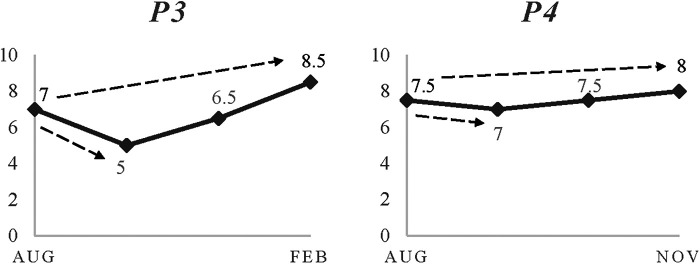
Two contrasting timelines showing impact and subsequent growth from an uncommon, planned challenge (living at digs).

Unsurprisingly, this was reflected on the subsequent perceived benefits and resulted growth from these challenges. As mentioned previously, the challenges that caused more emotional disturbance offered greater opportunities for growth. A prime example of this is the nuanced experiences of living at digs as part of a full-time registration programme (see Figure 5). P3 described benefits from the challenge:

I've learned a lot being away from home. Being independent is probably the main one. Also, because I've had to get through this, I learnt to focus on my main end goal of being a professional footballer. If something distracts me, like if I don't do well in a training session, I just remind myself of my end goal and focus on that instead.

While P3's account implied significant learning of new skills as a result of challenge, P4 identified benefits as increased confidence in his pre-existing skills to achieve his long-term goal:

My parents taught me to be independent and look after myself from a young age, so it's not particularly hard. I just take it as ‘I’m coming in for football, I'm sleeping here for football; everything's for my football’. Perhaps, it made me feel even stronger, knowing that I can deal with anything.

In short, while this challenge provided an ample opportunity for learning and growth for P3 (31% increase on his pre-challenge progression levels), for P4 provided more of a test and confirmation of existing skills (7%). Also, as clearly shown in Figure 5, P3 needed 6 months to reach his highest point following the challenge whereas P4 only needed 3 months.

**Figure 6:**
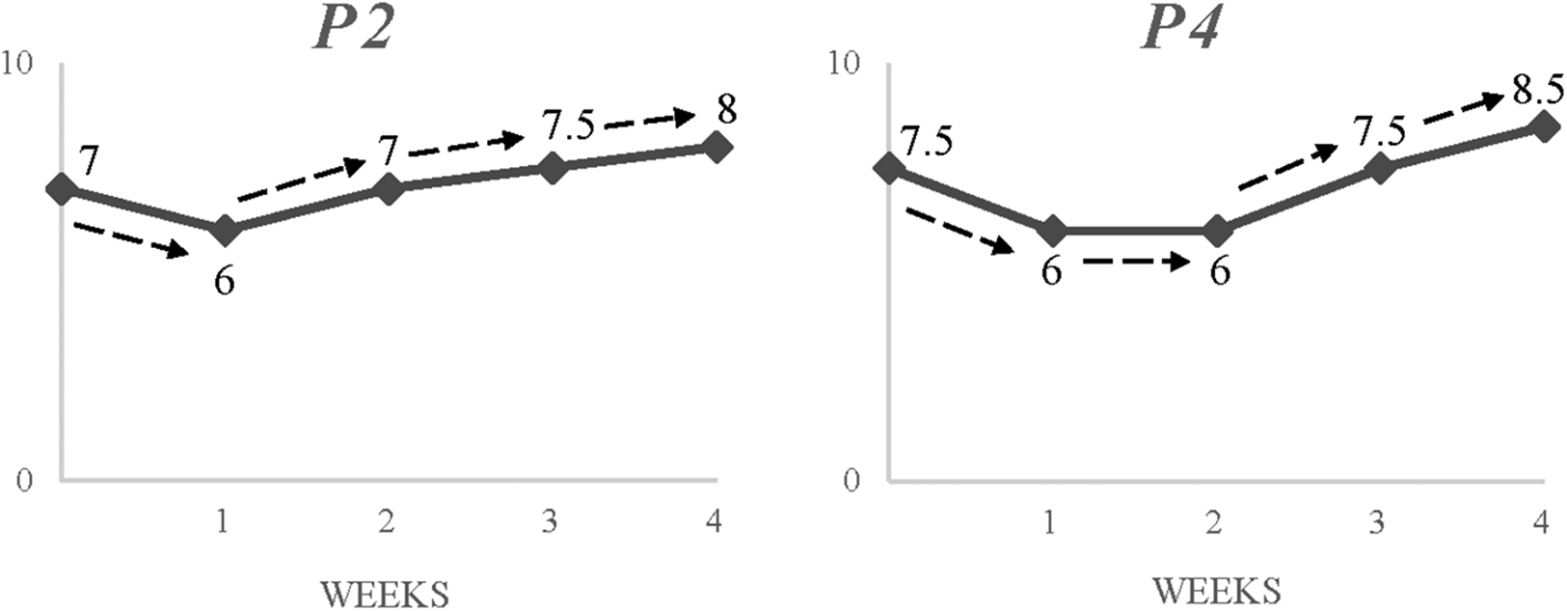
Two contrasting timelines showing rebound and growth from a common, unplanned challenge (underperformance).

Moreover, the process of navigating a challenge was also idiosyncratic. In fact, the data showed that participants utilised different coping skills or similar coping skills in different ways and recovered at different rates from similar challenges. Figure 6 illustrates the contrasting experiences of recovering and learning from a small period of underperformance. P4 stated:

I'd say myself more than anything on this one. I just took some time to think about how serious things are getting and how that can affect the things and people around me, people's perspectives of me. One little thing can change everything.

P4's account conveyed a sense of self-reliance to cope with his challenge. This involved allowing himself time to reflect and find meaning before exploring potential solutions, “It's about how you react, how you control your thoughts. I must keep my head high and pushing to the end every game, every training session”. Following a swift return to his normal pre-challenge progression levels after week 3, P4's account was able to identify positive changes by week 4, “Well, I could have just kept my mind to it, thinking about a previous mistake, but now I go into games thinking how well I’m going to do. I feel more in control”.

In contrast, P2 used social support which facilitated a quicker recovery to his pre-challenge progression levels (week 2):

You must ask for support. The coaches helped me out a lot, making me see this as a learning curve. They also give me advice and feedback. Seeing what you're doing wrong, correct them, giving yourself a better chance of getting in the team.

Although initial signs of growth were reported by week 3, P2 fully integrated positive changes following week 4, “I’m now working on my targets and just focusing on them. I am also being more reflective”. Whilst both participants reported post-challenge growth (14% and 13% respectively), yet the experience was individualised to them.

**Figure 7:**
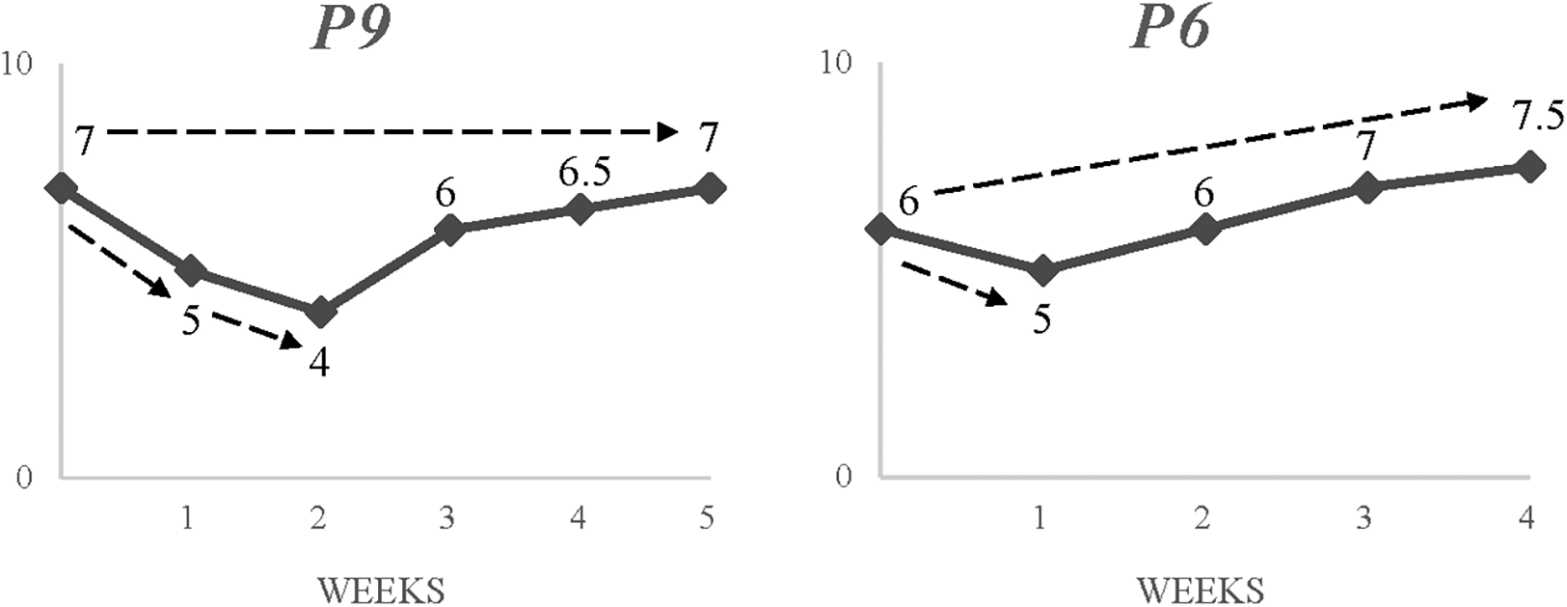
Two contrasting timelines showing impact and growth of planned vs. unplanned, uncommon challenge (playing out of position).

Whilst the influential role of individual characteristics in promoting learning from challenges was pervasive throughout the data, it is also the level of support and periodisation of challenge that seems crucial. Figure 7 shows how different versions of the same challenge (planned vs. unplanned) can lead to different outcomes. For example, P6 stated:

The coaches felt that I could learn a lot from it. It was tough, but they'd give me tips; what to do, how to move, what to look for. Their support throughout was great. So, if I had a good session, they might pull me aside and tell me stuff and if I struggled in the session they might come over and give me some constructive feedback.

P6's account clearly outlined a sense of clarity for the purpose of his challenge. His reference to support and regular feedback received throughout facilitated a sense of confidence in his ability to deal with the demands of the challenge. This is also supported by his perceived trajectory which depicts a stable progress through the challenge. In contrast, P9's statement conveys a sense of ambiguity regarding the scope of challenge, “I was worried; My confidence was sort of going down thinking “Maybe they don't like me as a central midfielder or someone else is doing better?”. Unlike P6, P9 experienced multiple drops in progression level. Notably, the period of challenge for P6 was 2 weeks whereas for P9 was 3 weeks. Also, P9's subsequent rebound was propelled by him seeking clarity after week 2, “Me and my dad had a chat with the coaches as I needed to know”. He then added, “They said my vision would improve playing as a centre back, seeing more passes, long and into the space. It’d also be an opportunity to lead my unit. This was helpful”.

Despite this, perceived developmental impact differed a lot with participant P6 reporting a 25% increase on his pre-challenge progression level after week 4 while P9 reported no growth at all. This is clearly supported by their subsequent reflections on the outcomes of the challenge. P9 stated, “Well, I managed to cope. But I didn't like it…It wasn't clear, and I didn't feel I learnt anything. Just to remind myself that I need to keep pushing even when things aren't great”. P6, on the other hand, was far more able to identify specific benefits accrued, “I improved my decision-making, like playing quicker under pressure and having 360 awareness. When to press and when to hold up. Things I’m using more now, playing in my main position”.

## Discussion

Given the established gap in research of tracking performers in real time, the aim of this study was to longitudinally track young performers' pathway as they negotiated and learnt from challenge across a 15-month period. The design of this study allowed us to explore the three targeted areas: (1) nature and timing of perceived challenges experienced by young footballers, (2) the perceived impact, benefits and subsequent development from these challenges, and (3) the idiosyncratic nature of these challenges.

For all participants, their pathway as an aspiring footballer was filled with recurrent challenging events causing repeated emotional disturbances (18). This further solidifies previous evidence that young performers do face challenges in sampling (15) and specialising (8) years, something which had previously gone undiscovered by retrospective studies [e.g., (7)]. Furthermore, the use of a mixed methods approach offered more information about the nature and impact of these challenges [cf. (45)]. In line with prior work (3, 7), the nature of the challenges recalled in this study were complex and multifaceted, including both sporting and non-sporting issues. Notably, however, this study adds more depth to the growing area of TD research (4, 8), by showing that these events can be further categorised into three areas, common/uncommon, planned/unplanned and group-focused/individual-focused challenges. Whilst most of the challenges were consistent amongst participants, some events appeared to be highly idiosyncratic showing that young performers' interpretations may vary. Furthermore, some challenges appeared to be age-related, suggesting that young performers may organically encounter specific challenges as they progress along the pathway. Crucially, these challenges constituted a combination of structured/carefully planned and unpredictable events. This suggests that the importance of deliberate challenge gains increasing recognition from TD stakeholders. Indeed, recent work has shed some light into how coaches use planned disruptions to facilitate growth in resilience (21).

Our findings support the suggestion that the emotional disturbance induced by challenges can facilitate learning and growth along the TD pathway (7, 8, 18, 46). Regardless of the level of emotional disturbance, however, the perceived progression level after these events was almost always greater than it was before. In fact, recent work posits that subsequent positive changes are due to experiencing something as “traumatic” (or significantly upsetting) rather than the severity of the cause itself (18). This is particularly relevant as previous retrospective studies (4, 7) might have been unable to capture such small doses of emotional disturbance which propelled growth, thus not generating a full picture of the role of developmental challenges in the context of TD.

Moreover, this study contributes to a deeper understanding of how challenge might impact development. Young performers progressed through a series of perceived stages in each challenge including *drop*, *rebound* and *growth*. To clarify, all initially reported a range of emotional responses to their challenge (drop), before reflecting on and altering their pre-challenge beliefs and approaches (rebound) thus leading to learning and positive changes (growth). Notably, these stages and mechanisms are consistent with previous findings in adversity-related growth research (14, 47, 48). Indeed, the ability to employ a staged approach, engage in cognitive processing, tolerate distress, find meaning, undertake belief change, and adapt one's behaviours, have been linked with constructive growth (49, 50). Crucially, the detailed reflection promoted by the perceived effect of emotional disturbance appears to be central to the subsequent learning process (51, 52).

Notably, successful navigation of these events was underpinned by the young performer's ability to apply various coping skills throughout the three stages from drop to growth. Although it is beyond the scope of this paper to delve into the different types of coping skills reported, these included mental skills (e.g., ability to focus, goal setting), social support skills (e.g., mobilising and using social support networks) and learning skills (e.g., reflections, making sense and identify learning from challenges). The influential role of these coping skills in facilitating optimal benefits from challenge is well established [e.g., (53–57)].

These common skills notwithstanding, participants reported a unique development journey, further denoting the idiosyncratic nature of the TD pathway (7). Whilst the types of challenges experienced appeared to be common amongst the young performers, the timing, perceived impact, scale and, ultimately, consequential developmental impact of these varied greatly. More specifically, young performers showed significant variability in their initial emotional responses to similar events. Notably, a number of individual factors appeared to underpin these different nuances in responses (17, 18). Akin to recent evidence (8), such responses were dependent on the initial interpretation of the impact of the challenge. To clarify, these events involved experiences of either physical, social, emotional and/or cognitive challenges, with participants' interpretations of their impact being varied.

Moreover, different perceptions in terms of the scale (i.e., difficulty) of the challenge appeared to be influenced by prior individual experiences and the possession of coping skills. In fact, the notion of intrapersonal growth after challenge is well-reported in previous work (22, 58). Therefore, given the individualised nature of early experiences and skills acquired both along the TD (7, 59) and pre-entry (15), young performers may exhibit different emotional reactions to similar events based on these. Resultantly, while a challenge may serve as an opportunity to test and grow for one, for someone else may offer little new learning. Importantly, the use of these coping skills and rate at which they experience rebound, and growth is also individualised. In short, to cope with similar challenges, they may utilise different skills or similar skills in a different way (7).

Findings from this study would also suggest that the provision of challenge alone may not be sufficient for progression, and may even derail performers' progression (60, 61), unless it comes with support relative to the scale of the challenge. Indeed, recent evidence has shown that high levels of challenge (62) causing negative emotional states repeatedly, with negative or no feedback may have adverse effects, unless one is already in possession of strong coping skills and a non-sport related support network (18). Critically, this alludes to recent calls for preparation for/debrief of challenge (63, 64) in a coherent manner (65) and timing/monitoring of its impact (6) to ensure optimum outcomes.

## Implications and next steps

Reflecting the above, we suggest there are several practical implications for TD settings. We believe the findings of this study provide a novel insight into the nature of challenges, including the types and timing occurred, as well the interpretation of these challenges by young performers, including the perceived scale, emotional impact and consequential developmental impact of them. Specifically, young athletes appear to develop in a much more structured ways whilst on the pathway than pre-entry [cf. (15)]. Indeed, the coaches placed particular emphasis on the deployment of planned obstacles in order to facilitate learning and growth. However, evidence in this paper shows that not every challenge is created equal as young performers experienced similar events differently. It becomes evident from our data that the nature, timing, scale and structured support around the challenge are as important as the skillset. Therefore, we would challenge uniform approaches to the deployment of structured challenges given the idiosyncratic nature of negotiating challenges on the TD pathway. This emphasises the critical consideration of how much challenge, of what kind, when and how dealt with will maximise benefits.

Therefore, as a means of effective individualisation, practitioners may significantly benefit from systematically assessing the young performers' coping skills and prior experiences before and after entry [cf. (15)], and to keep monitoring their needs as they continue to develop along the pathway. Also, practitioners should be conscious of current sport and non-sports-related stressors and psycho-emotional states of the athletes (62). This information will enable these practitioners, supported by all stakeholders, to make informed decisions and subsequently deploy appropriate challenge and support (19) to maximise young athletes' development. Taken collectively, young performers must be adequately prepared for the challenge (i.e., possession of coping skills) whose design must offer appropriate levels of emotional disturbance to test their skillset (18). Crucially, however, these challenges should be realistic, timely, and appropriate to the needs of the performer. There is a need for clarity in the message and support received at each level including priming, necessary feedback, debrief and reflection opportunities that appear to be crucial for promoting adaptive response to challenge (56).

Extending on these findings from an athlete perspective, it would be beneficial to triangulate perceptions from the wider network (coaches and parents/guardians), thus distinguishing between actual (indicated as growth identified by both performer and stakeholders) and reported (personal perceptions of performer) growth. In fact, several theories have advocated this. Cho and Park (66) in their review on growth after trauma suggest that there are undisputable differences between actual and reported growth, with reported growth following adversity is not necessarily linked to actual changes. Given that the process of talent development is complex and multi-factorial (67) and the relevance of ecological-dynamics rationale in current research landscape (68), triangulation research seems to be an imperative lens to explore. Also, given the dynamic development of brain during adolescence (69) along with the idiosyncratic nature of early experiences (15), future research may explore the different responses to challenges between age groups.

Given the nature of the study design, it must be acknowledged that there are some limitations. By asking participants to reflect on their unique experiences of challenges, there is a risk of self-presentation. Additionally, all participants were “highfliers” within the academy and it may be that a less positive picture would have emerged if we examined their less successful counterparts. Also, the sample size was relatively small and limited within the context of professional academy football. Another limitation was the sole focus on male athletes. Therefore, future research may employ similar methodology to explore player experiences with female athletes. These limitations may raise concerns around the transferability of our findings to other team and individual sports. Transferability, however, stresses the importance of critical consideration of applicability across different domains whilst challenging the view that findings offer generalizability (70). Therefore, and in accordance with our pragmatic philosophy where methods are purposefully designed to allow transferability across contexts, we suggest that practitioners and researchers who have interacted with this paper critically consider our findings within their performance domain.

In conclusion, this study shows that growth is experienced as a part of a combination of carefully planned process and a series of emergent occurrences. It also provides a unique lens on the idiosyncratic nature of how young performers interpret challenges and the subsequent stages they go through in order to negotiate these challenges and experience growth. To this end, developmental impact appears to be influenced by an interaction between individual characteristics and context of challenge including coping skills, interpretation, timing and integration of support. Therefore, practitioners should systematically assess and monitor the individual skillset and needs. This information should provide the platform for TD stakeholders to individualise young athletes' pathway through deliberate preparation processes (teaching, priming), reflection and feedback processes (tweaking) and, ultimately, deployment and periodisation of developmentally appropriate challenges along the TD pathway.

## Data Availability

The original contributions presented in the study are included in the article/Supplementary Material, further inquiries can be directed to the corresponding author.
